# Natural Killer Cells as a Further Insight into the Course of Chronic Obstructive Pulmonary Disease

**DOI:** 10.3390/biomedicines12020419

**Published:** 2024-02-11

**Authors:** Beata Brajer-Luftmann, Tomasz Trafas, Marta Stelmach-Mardas, Weronika Bendowska, Tomasz Piorunek, Marcin Grabicki, Mariusz Kaczmarek

**Affiliations:** 1 Department of Pulmonology, Allergology and Pulmonary Oncology, Poznan University of Medical Sciences, Szamarzewskiego 84 Street, 60-569 Poznan, Poland; ttrafas@ump.edu.pl (T.T.); t_piorun@op.pl (T.P.); grabicki@ump.edu.pl (M.G.); 2 Department of Treatment of Obesity, Metabolic Disorders and Clinical Dietetics, Poznan University of Medical Sciences, Szamarzewskiego 84 Street, 61-569 Poznan, Poland; stelmach@ump.edu.pl; 3 Department of Immunology, Poznan University of Medical Sciences, Rokietnicka 5 Street, 61-806 Poznan, Poland; weronika_michalska@wp.pl; 4 Department of Cancer Immunology, Poznan University of Medical Sciences, Garbary 15 Street, 61-866 Poznan, Poland; markacz@ump.edu.pl; 5 Gene Therapy Laboratory, Department of Cancer Diagnostics and Immunology, Greater Poland Cancer Centre, Garbary 15 Street, 61-866 Poznan, Poland

**Keywords:** BALF, NK, COPD, immune response, pulmonary function, aging

## Abstract

The role of natural killer (NK) cells in chronic obstructive pulmonary disease (COPD) pathogenesis has been discussed but is not yet clearly understood. This current study aimed to evaluate the associations between immunophenotypes, degrees of maturity, and the expression level of functional receptors of NK cells in the lung environment present in bronchoalveolar lavage fluid (BALF), and an attempt was made to determine their relationship in the course and progression of COPD. A total of 15 COPD patients and 14 healthy smokers were included. The clinical parameters of COPD were evaluated. In both groups, NK cells using monoclonal antibodies directly conjugated with fluorochromes in flow cytometry were assessed in the peripheral blood. Additionally, NK cells using the same method were assessed in BALF in the COPD subgroup. The blood’s NK cells differed from the estimated group’s maturity and receptor expression. Functional receptors CD158b+, CD314+, and CD336+ expressed by NK cells were significantly interlinked with age, RV, TLC, 6MWT, smoking, and the number of exacerbations. These results confirm the essential role of NK cells in COPD pathogenesis. Additionally, the relationship between clinical parameters and NK cell expression may indicate its participation in the disease progression and exacerbation and allow for a better understanding of NK cell biology in COPD.

## 1. Introduction

Chronic Obstructive Pulmonary Disease (COPD) is a heterogeneous, progressive lung disease resulting from interactions between the genetic background of the host, environmental risk factors, and aging processes over the lifetime [1,2,3,4]. Based on the Global Burden of Disease Study [5], it can be estimated that globally, there are around 3 million deaths annually due to COPD, with a prognosis of increasing up to 5.4 million by 2060. Smoking status is the most important factor responsible for COPD in low-income countries, whereas the aging process plays a major role in highly developed countries [3]. Exposure to harmful particles, including tobacco smoke, leads to impaired mucociliary clearance and damage to the epithelial barrier [1]. Damage to the respiratory epithelium increases the migration of inflammatory cells [1,2,3], and inflammation in the respiratory tract is initiated [2]. As the disease progresses, inflammatory cells activated by tobacco smoke secrete inflammatory and chemotactic mediators, intensifying a modification of the normal inflammatory response to harmful agents, i.e., cigarette smoke, which may continue even after smoking cessation [2,4]. The pathological lesions underlying COPD development include the airways, lung parenchyma, and pulmonary vessels [4]. In these locations, an increased percentage of immune cells has been observed, including macrophages, stimulated neutrophils, and various subpopulations of lymphocytes. [2,3].

The percentage of natural killer (NK) cells in the lungs, including three subpopulations: CD56dimCD16+, CD56dimCD16−, and CD56brightCD16−, slightly exceeds that observed in peripheral blood and ranges from 5 to 20% of lymphocytes [6]. Each of them expresses three markers of residency differentially. Most NK cells present in the lung are circulating cells that display a terminally differentiated phenotype and belong to the CD56dimCD16+ population, unlike resident NK cells that express CD69, CD49a, and/or CD103 antigens. Almost all resident NK cells show the less mature CD56brightCD16− or, to a lesser extent, CD56dimCD16− immunophenotype. Among them, triple-positive CD49a+CD69+CD103+ constitute less than 3% of the total number of NK cells in the lungs [6,7,8]. The NK cells’ survival is supported by bronchial epithelial cells that spontaneously release IL-15 [9]. Under physiological conditions, the NK cells’ cytotoxic function in the lung is suppressed [10]. Upon inflammatory stimulation, as in COPD, NK cells migrate from the blood to the lungs, where they may participate in COPD pathobiology [3,9]. Thus, the extent of NK cells’ activation in COPD depends on the balance between pro-inflammatory and regulatory factors [11,12,13].

Currently, for COPD, it is crucial to understand the nature of the disease and the risk factors for its progression. The homeostasis of immune system and role in pathogenesis of COPD is driven by NK cells; however, the further link with clinical parameters still needs to be clarified. This current study aimed to evaluate the associations between immunophenotypes, degrees of maturity, and the expression level of functional receptors of NK cells in the lung environment present in BALF and attempted to determine their relationship with the course and progression of COPD.

## 2. Materials and Methods

### 2.1. Study Design

This was a prospective study. COPD patients and control volunteers were recruited in the outpatient clinic of the Department of Pulmonology, Allergology, and Pulmonary Oncology, Poznan University of Medical Sciences (Poland).

The study protocol was approved by the Bioethical Committee at Poznan University of Medical Science (No: 741/10). All enrolled participants provided written informed consent before entering the study.

### 2.2. Study Population

Fifteen stable COPD patients (10 males and 5 females) were studied. The inclusion criteria for the study were as follows: current or former smokers with normal body weight, COPD patients diagnosed according to the GOLD 2018 criteria [14] if their FEV1/FVC ratio after inhalation of 400 µg of salbutamol was less than 0.7, and with a clinical indication for VBF (e.g., chronic cough, difficulty in expectorating sputum). A total of 14 healthy volunteers (9 males and 5 females) with smoking history (current or ex-smokers) were matched, as far as possible, for gender, age, and number of pack years with COPD patients. In the whole COPD group, the number of exacerbations per year was recorded; pulmonary function tests (PFTs), six-minute walking test (6MWT) [15], and arterial blood gas analyses were performed. The 6 min walking test was performed in a closed room in a corridor with a minimum length of 30 m. The turning point was marked with a post, the starting line was drawn, and the distance was divided into 3 m sections marked with points on the ground. After resting in a sitting position for a minimum of 10 min, a minimum of 2 h after physical exercises and measurements of oxygen saturation and heart rate, and after recording the degree of shortness of breath on the Borg scale, the patients were asked to walk as far as possible at their own pace for 6 min. The patients could stop if they felt tired. After completing the test, the patients were again asked to assess shortness of breath on the Borg scale, and the pulse and oxygen saturation were measured; the distance passed was also recorded [15]. Blood samples for gas analysis (1.5 mL to the self-filling syringe containing 60 IU dry heparin) were obtained from the patient’s radial artery after 15 min of resting in the sitting position (Simens RAPIDLab 1265, Camberley, UK, 2012) [16]. Additionally, 9 mL peripheral blood samples were taken for cytometric analysis in the study groups and collected in tubes containing EDTA. Some COPD patients underwent videobronchofiberoscopy (VBF) due to clinical symptoms following British Thoracic Society guidelines [17,18] in the Bronchoscopy Laboratory, which is the university unit. All procedures were conducted according to good laboratory and diagnostic practice per the Helsinki Declaration.

The following exclusion criteria were taken into account: other pulmonary disorders (e.g., asthma, history of tuberculosis), a clinical suspicion of current infection, an allergy, a pulmonary thromboembolism, and interstitial diseases. COPD patients were also excluded if they had an exacerbation and/or received antibiotics or corticosteroids (oral and/or inhaled) within the previous six weeks before inclusion in the study.

### 2.3. Pulmonary Function Tests

The selected tests (spirometry, body plethysmography, and the diffusion capacity of carbon monoxide-DLCO) with the use of Master Screen Body/Diffusion Jaeger device (Erich Jaeger GmbH, Würzburg, Germany) were performed. The American Thoracic Society/European Respiratory Society guidelines were followed [19]. Spirometry was conducted 15–30 min after the inhaling of 400 µg of salbutamol. Results were presented as % predicted according to the Global Lung Function Initiative (GLI) references [20]. Airflow limitation was defined as a post-bronchodilator FEV1/FVC ratio < 0.70 [18].

### 2.4. Videobronchofiberoscopy and Bronchoalveolar Lavage Fluid (BALF)

VBFs were performed by an experienced bronchoscopist using a flexible video bronchoscope (BF-H190 Olympus, Tokyo, Japan). The procedures were carried out according to the British Thoracic Society guidelines, including information on indications and contraindications [16,17]. During the VBF, bronchoalveolar lavage fluid (BALF) samples were collected according to the international guidelines [21,22,23]. The tip of VBF was wedged in the segmental or subsegmental bronchus of the middle lobe. The bronchi were rinsed with 50 mL of 0.9% sterile saline solution at 37 °C and aspirated. Then, two more portions of 50 mL of 0.9% sterile saline at 37 °C were poured and aspirated similarly. The amount of fluid recovered from bronchoalveolar lavage was from 20 to 40 mL [21,22,23].

### 2.5. Immunophenotypic Assessment

The immunophenotypic evaluation of fresh, unfixed cells from BALF and peripheral blood (PB) was performed with the use of a flow cytometer. During the analysis of surface markers characteristically used for NK cells, monoclonal antibodies directly conjugated with fluorochromes were used (Table 1).

BALF samples were prepared for analysis as follows: first, they were filtered through sections of sterile gauze. The cell suspensions were centrifuged for 10 min at 1800 rpm. Next, cells from BALF and whole peripheral blood previously collected in tubes with anticoagulant (EDTA) were added directly in a volume of 100 µL to cytometric polypropylene reaction tubes, to which 5 µL of monoclonal antibodies directed against selected antigens had been previously added. During the tests, negative controls consisting of cells of the tested samples to which no antigen-specific antibodies were added, as well as appropriately configured isotype controls. Samples of the tested material were mixed thoroughly with antibodies and then incubated for 15 min in the dark at room temperature. After this time, 500 µL of commercially available lysis buffer (Becton Dickinson, Franklin Lake, NJ, USA) was added to the tubes to eliminate erythrocytes and consolidate the bonds between antigens and their corresponding antibodies. The lysis process was carried out during subsequent incubation for 10 min at room temperature. Phosphate-buffered saline (PBS) was added to the tubes to inhibit lysis. Lysed samples were finally washed out twice through centrifugation for 5 min at 1500 rpm and resuspended in 200 µL of PBS for acquisition using a BD FACSAria Cell Sorter flow cytometer (BD Biosciences, San Jose, CA, USA). The BD FACSDiva Software v. 6.1.2 (BD Biosciences, San Jose, CA, USA) compatible with the cytometer was used for analysis. During the analyses, the percentage of cells expressing the tested differentiation markers was determined (% of positive cells), and the level of expression of the tested antigens was determined based on the mean fluorescence intensity (MFI) parameter.

### 2.6. Statistical Analysis

The Shapiro–Wilk test was used to check the normality of the distribution. Due to the small study group and the lack of a normal distribution, the data were presented as the median and interquartile range. The Mann–Whitney test was used to analyze the differences between the compared groups, i.e., the percentage of NK cells among all leukocytes and lymphocytes, and expression markers in peripheral blood (PB) and BALF and PB in COPD patients, and in PB in the control group. The Spearman correlation coefficient was used to assess the presence of a relationship between NK cells and clinical parameters. Statistically significant differences were defined as *p* < 0.05. All calculations were carried out using Statistica 13 software (TIBICO Software Inc., Palo Alto, CA, USA).

## 3. Results

### 3.1. Subjects’ Characteristics

The study population was characterized as shown in Table 2. All study participants were current or former smokers, characterized by a normal weight. Additionally, nine patients with COPD underwent VBF for clinical reasons (chronic cough, difficulties with expectorating sputum).

### 3.2. Immunophenotypic Evaluation of NK Cells

**Figure 1:**
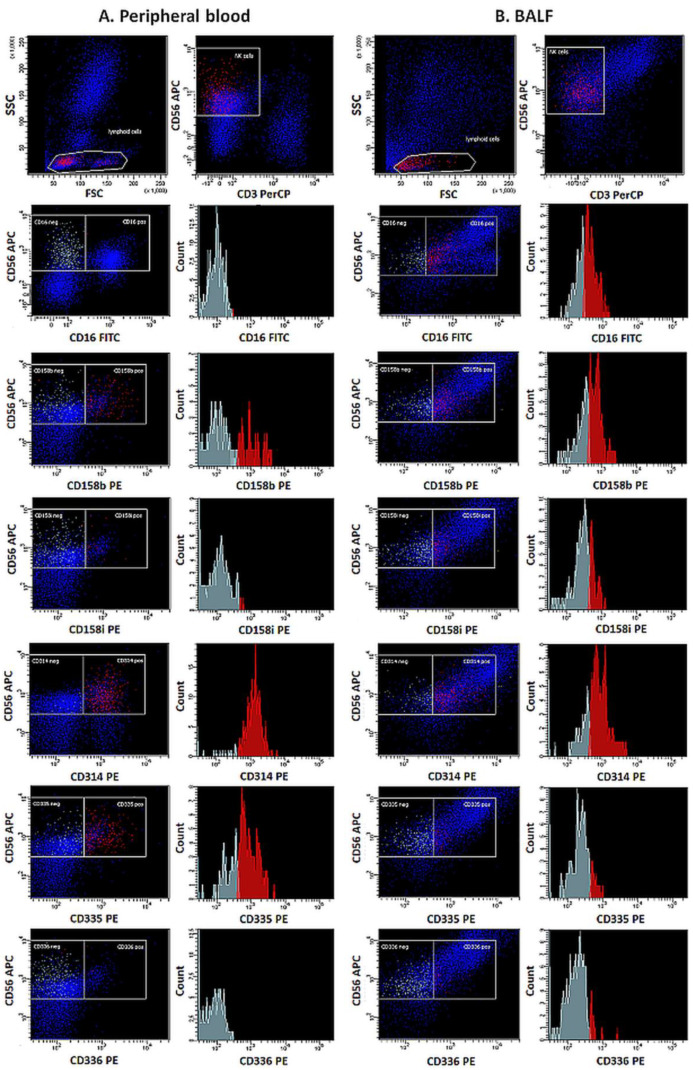
Expression of functional receptors on NK cells in peripheral blood (**A**) and BALF (**B**) in exemplary samples.

The presence of NK cells in the assessed materials was demonstrated based on their morphological parameters, size (Forward Scatter Channel; FSC), granularity (Side Scatter Channel; SSC), and characteristic immunophenotypic pattern. In the first stage of the cytometric assessment, areas distinct from lymphocytes were outlined on scattergram plots based on the FSC and SSC values. Then, from the indicated location, NK cells were characterized by determining the expression of characteristic differentiation markers CD56+ and CD16+ in the absence of CD3− (Figure 1).

**Figure 2:**
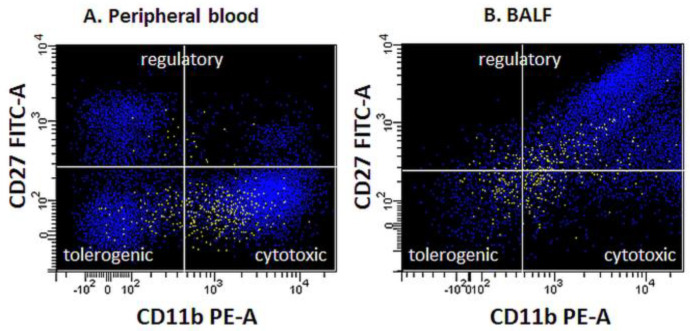
Functional profiles (maturity state) of NK cells in peripheral blood (**A**) and BALF (**B**) in exemplary samples.

The maturity of the NK cell population evaluated was determined based on the expression of a combination of antigens CD11b and CD27. The percentages of NK cells expressing CD158b, CD158i, CD314, CD335, and CD336 receptors were also determined, and the level of expression of these receptors was assessed by measuring the Mean Fluorescence Intensity (MFI) (Figure 2).

### 3.3. The Percentage of NK Cells

The percentage of NK cells in peripheral blood (PB), both among all leukocytes and lymphocytes, and in BALF, was assessed in COPD patients. A significant difference between the percentage of NK cells in BALF and PB was visible—in the total sample and in the lymphocyte population. The percentage of NK in the total sample cells in BALF was higher than that in PB, but the percentage of NK cells calculated among lymphocytes assessed in BALF was lower than that in PB (Table 3).

Similarly, the expression of CD11c and CD27 antigens as maturation NK cell markers and the expression of CD158b, CD158i, CD314, CD335, and CD336 molecules as functional receptors were evaluated in PB and BALF using both the percentage of positive cells and mean fluorescence intensity (MFI), which represents the level of antigen expression. The study results revealed the differences between assessed samples obtained from COPD patients and the control group (Table 4).

### 3.4. Association between NK Cells, Their Receptors, Maturity Markers, and Clinical Parameters

An association between the percentage of NK cells, calculated in terms of the total sample, as well as among the lymphocyte population, their maturity markers and their functional receptors expression level, and clinical features, were evaluated.

**Figure 3:**
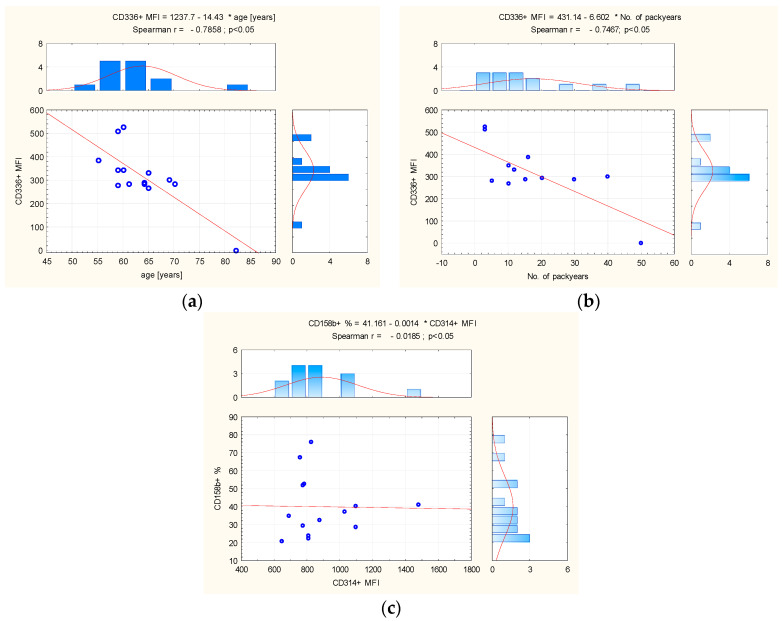
(**a**) The correlation between age and CD336+ MFI, and (**b**) between number of pack years and (**c**) CD314+ MFI and CD158b+ in control group peripheral blood. Abbreviation: MFI = Mean Fluorescence Intensity. The red line = graphical presentation of Spearman correlation.

In peripheral blood from the control group, the statistical analysis showed a significant positive association between number of pack years and the level of expression (MFI) CD314+ and (r = 0.7323; *p* < 0.01), and CD158b+ (r = 0.5907; *p* = 0.03). In this group, a significant negative relationship was also observed between age and CD336+ MFI (r = −0.5871; *p* < 0.03) (Figure 3).

**Figure 4:**
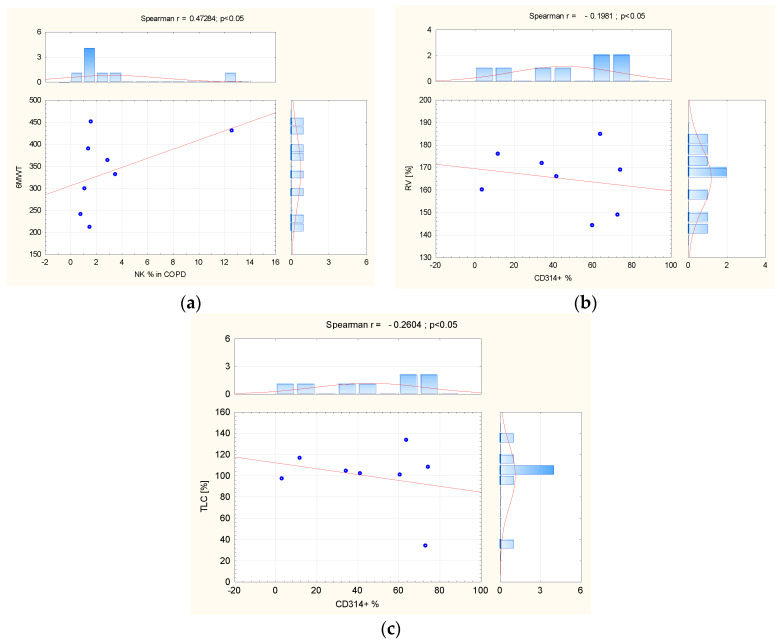
(**a**) The correlation between NK cells’ percentage in COPD peripheral blood and 6MWT; the correlation (**b**) between CD 314 receptors and RV% predicted and (**c**) TLC% predicted in the same group. Abbreviation: COPD = Chronic Obstructive Pulmonary Disease, NK = natural killer, TLC = total lung capacity, RV = residual volume. The red line = graphical presentation of Spearman correlation.

In peripheral blood from COPD patients, the percentage of NK cells, calculated among lymphocytes, was positively correlated with 6MWT (r = 0.5259; *p* = 0.04). In peripheral blood of COPD patients as well, significant positive correlations were observed between the percentage of CD314+ (NKG2D) receptors expressed on the CD56+ bright subpopulation and residual volume (RV)% pred. (r = 0.5780; *p* = 0.03), and total lung capacity (TLC)% pred. (r = 0.7195; *p* < 0.01) (Figure 4).

**Figure 5:**
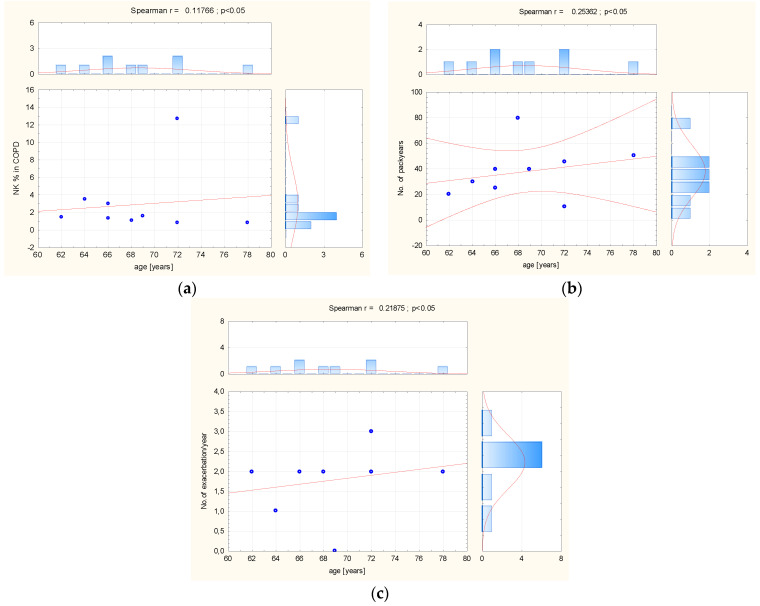
(**a**) The correlation between age, (**b**) number of pack years, (**c**) number of exacerbation, and NK cells pattern in BALF taken from COPD patients. Abbreviation: MFI = Mean Fluorescence Intensity, No = number. The red line = graphical presentation of Spearman correlation.

In BALF taken from COPD patients, statistical analysis revealed a significant negative relationship between NK percentage and the number of pack years (r = −0.8033; *p* < 0.01), and a positive association between the number of pack years and CD27−/CD11b+ cells (r = 0.7699; *p* < 0.02). The study results showed significant negative correlation between age and CD314+ MFI expression (r = −0.7899; *p* = 0.01), and also CD158b+ MFI (r = −0.9244; *p* < 0.01). In this group, a significant negative relationship was observed between the number of exacerbation per year and CD336+ percentage (r =−0.6832; *p* = 0.04) and CD158b+ percentage (r = −0.7327; *p* = 0.02) (Figure 5).

## 4. Discussion

One of the hallmarks of the chronic inflammatory process found in the lung tissue and airway lumen of COPD patients is the activation of the innate immune system, defined as an increased number of innate immune cells such as neutrophils, macrophages, mature dendritic cells, and natural killer (NK) cells [3,12]. NK cells are the human body’s first defense against infections and cancers [24] and may take part in maintaining immune homeostasis and the pathogenesis of COPD [12,25,26]. The results of our work show that NK cells found in COPD patients differ in maturity and the presence of surface receptors. The specific panel of NK cells depends not only on the presence of COPD but also on the age of the study patients included. Moreover, through the association with the residual volume and total lung capacity of COPD, the results sof the current study support the hypothesis of the influence of NK cells on structural changes.

The frequency of the occurrence of NK cells may differ between patients with COPD, healthy smokers, and healthy non-smokers [27,28,29,30]. As a result of cigarette smoking, conditions within innate immunity change. The percentage of NK cells and accompanying macrophages increases, while the percentage of dendritic cells decreases. Despite the observed increased numbers, the NK cells of cigarette smokers are characterized by impaired functional potential, manifested by reduced cytolytic function, both in terms of cellular and antibody-dependent cytotoxicity. NK cells under the influence of cigarette smoke exposure have a reduced expression of the CD16 molecule and reduced ability to synthesize TNFα and perforin [31]. At the same time, the possibility of a beneficial effect of smoking cessation in the short term is indicated not only on clinical indicators and respiratory function, in terms of airway obstruction parameters, but also on metabolic indicators as well as the reversal of negative indicators in terms of immunological exponents, including NK cell activity [32,33,34]. However, the indication of the reversibility of the functional status of NK cells in COPD patients who quit smoking has not yet been clearly established. The study of Chen et al. [35] showed higher percentages of NK and NKT cells in PB COPD patients than in healthy non-smokers. On the other hand, Urbanowicz et al. [36] showed that the proportion of peripheral blood NKT-like (CD56+CD3+) cells in smokers with COPD was significantly lower than in healthy smokers. The results of another study by the same group of researchers [27] showed that in induced sputum, the proportion of NK cells and NKT-like cells in smokers with COPD was significantly higher than in healthy smokers and non-smoking healthy subjects. Interestingly, Andersson et al. [29] indicated that PB NK cell frequency is not differentiated between COPD patients and healthy smokers and non-smokers. Hodge et al. [37] confirmed that there were no differences between the percentage of PB NK cells in healthy non-smokers, smokers, current smokers COPD, and ex-smokers COPD. The present study also found no differences between percentages of NK cells in PB from COPD and healthy smokers. However, in the group of COPD patients, the percentage of NK cells in BALF was significantly higher than in PB and did not depend on the number of pack years. Interestingly, in BALF taken from COPD patients, smoking status was negatively correlated with NK cell percentage and positively correlated with CD27−/CD11c+ expression. However, in PB from the control group, smoking revealed a positive correlation with CD314+ expression and CD158b+ receptors. The results cited above and our studies are contradictory regarding the number of NK cells and their features in the PB and BALF of COPD patients and healthy smokers. It may be connected with differences in studies protocols and in the characteristics of included study groups.

A noteworthy result of our study is the difference in the maturity of NK cells in the PB of COPD patients and healthy smokers, and between BALF and PB in the COPD group. NK cells, described in terms of immunophenotype as CD3−CD56+, are divided into two main subpopulations, differing in their maturity and functional status: CD56brightCD16− and CD56dimCD16+ [7]. CD56dimCD16+ NK cells are characterized by strong cytotoxic activity, the ability to induce antibody-dependent cellular cytotoxicity (ADCC), and a high expression of the immunoglobulin-like receptor (KIR), while CD56brightCD16− NK cells are less mature, their main function is the synthesis and secretion of cytokines, and they do not express the KIR receptor [7,38,39]. Silva et al. [40] and Fu et al. [41], in their studies, provided evidence that the expression of CD27 may reflect the distinct populations of human NK cells and may be connected with their immature developmental stage and the best ability to secrete cytokines. Similarly, the expression of CD11b on NK cells might be connected with their immature stages and responsible for their high cytolytic function [40]. Freeman et al. [42] and Finch et al. [43] revealed that NK cells from COPD patients are more prone to damaged autologous lung epithelial cells than NK cells from healthy subjects. In our study, we observed that more immature (CD11b−) NK cells with regulatory function were present in the PB from COPD patients than in the control group and independently of CD27 expression. No explanation for this phenomenon was found in the literature known to the authors. It may be connected with systemic and local chronic inflammation in COPD. Do those immature NK cells develop in lymphoid tissue, recruit to the circulatory system, and stay in PB in healthy smokers? And on the other hand, do mature NK cells migrate to the lung microenvironment and play a cytolytic function? Do immature ones stay in PB, taking part in regulatory role in COPD? There are more questions than answers about this process, and further work is needed to help understand this phenomenon.

NK cell regulation and their function depend on the various inhibitory and activating receptors that belong to natural cytotoxicity receptors or KIRs [44], and the activating NKG2D receptor (CD314) [45]. The interesting feature that our study reveals is that the phenotype of NK cells in PB in COPD showed increased expression of CD158b, CD158i, and CD314 belonging to regulatory receptors. Our study demonstrated that the expression of CD314 and CD158 on the NK cells increases with the number of pack years. Interestingly, this relationship only concerned NK cells isolated from the PB of healthy smokers. One of the known stimulation factors in the immune system is cigarette smoking, which may influence local and systemic inflammation and may be responsible for cellular damage [46,47]. The CD158 receptor has activating and inhibitory potential, influencing the development and activity of NK cells by interacting with MHC I [24]. The interaction of CD158b with specific HLA-C antigens on the target cell (e.g., alleles HLA-Cw1, HLA-Cw3, HLA-Cw7) inhibits cytotoxicity, prevents lysis, and targets cell death. Interactions between KIR and MHC class I are thought to be important in regulating NK cell and T cell activity following antigen stimulation [48,49]. In our study, we also notice increased percentage and expression of CD158i and a decreased percentage of CD314 in BALF in comparison to the PB in the COPD group. This phenomenon may suggest that NK cells in the lung microenvironment, where intense inflammation takes place, in COPD patients may show an increased state of activation, manifested by an increased share of cells expressing functional receptors.

It is known that systemic inflammation increases with age. During this time, a decrease in the cytotoxic function and cytokine secretion capacity of NK cells and a reduction in the percentage of naïve lymphocytes are observed in peripheral blood. Moreover, the immunophenotype of NK cells is shifted towards the mature CD16+CD56bright state [50]. Shehata et al. [51] and Chiu et al. [52], in their studies, demonstrated the functional plasticity of NK cells, depending on the environment in which they were present. The introduction of NK cells obtained from young mice into old recipients resulted in their functional impairment; however, NK cells from older mice transferred to young recipients regained their functions. These findings demonstrate that the age-associated alterations of NK cell activity can be at least partially attributed to the aged environment. These results reveal that the age-associated alterations of NK cell activity may be partially connected to the aged environment [50]. Our study did not show any relationship between the percentage of NK cells in both assessed groups. However, in the group of healthy smokers, it was noticed that the amount of CD336 receptors on the surface of NK cells significantly decreased with age. The CD336 receptor is involved in the activation of the cytotoxic reaction [53]. In COPD patients, the relationship between age and NK cell activity was observed in the current study only in the lung microenvironment. NK cells in this group of patients showed reduced CD314 MFI expression and CD 158b+ MFI with age. These molecules belong to the activating receptors group on the NK surface. Therefore, their reduced presence may suggest an impairment in the ability of this cell population to mount an immediate cytotoxic response in aging, especially in the lung microenvironment [53]. In this way, the COPD patient may be more susceptible to harmful particles inhaled.

Based on previous studies, it can be assumed that the functionality of NK cells may change as the COPD progresses [26,36,42,43,54,55]. The CD56-mediated cytotoxicity of NK cells may increase with the severity of obstruction, as measured by predicted FEV1 [43]. Freeman et al. [42] showed that greater expression by autologous lung epithelial cells of the NKG2D ligands, MICA/MICB, but not expression by lung CD56+ cells of the activating receptor NKG2D, was inversely correlated with the predicted FEV1%. Pascual-Guardia et al. [56] observed that the proportion of NK cells was inversely correlated to the FVC but did not differ significantly with TLC and the distance obtained in the 6 min walking test. In the current study, the COPD population was assessed using PFT and 6MWT distance. Contrary to the research cited above, we noticed that the percentage of NK cells in PB of COPD patients increased with longer distances on 6MWT. This may suggest that a healthier population of COPD patients has a more significant number of circulating NK cells. During COPD progression, related to the shorter 6MWT distance, the NK cells may move from circulation to the lung microenvironment and play their role there. We also observed that CD314 expression increased with RV growth and TLC. In the available literature, we did not find such a relationship. This phenomenon may mean that NK cell cytotoxic activity may increase with the degree of lung hyperinflation. These results indicate that NK cells may play an essential role in COPD progression and support the thesis that NK cells may participate in emphysema development.

COPD exacerbations are often associated with increased local and systemic inflammation caused by respiratory infection, pollution, or other lung damage [57]. Hong et al. [58] indicated that the NK cell ratio was higher in the acute exacerbation (AE) COPD group than with respiratory infectious disease, and those with respiratory non-infectious disease group. Ryu et al. [59] identified 890, 675, and 3217 genes associated with a history of acute exacerbation of COPD (AECOPD), persistent exacerbations, and prospective exacerbation rate, respectively. In COPDGene, the number of prospective exacerbations in patients with COPD was negatively associated with circulating resting NK cells [59]. Another study showed that NK cells from COPD patients were not responsive to *S. pneumoniae*, suggesting functional defects of NK cells in COPD patients [60]. The increased level of PB NKG2C+ NK cells was closely associated with the number of exacerbations, suggesting a potential role in predicting COPD exacerbations [56]. The results of our study correspond to those cited above. As the number of exacerbations increased, we observed a decreased expression of CD158b and CD314 lung microenvironment by NK cell receptors. The reduced expression of the above receptors may suggest the lung NK cell population’s limited ability to immediately perform cytotoxic response and transform it into a regulatory function. It may be assumed that during an acute exacerbation of COPD, some of the NK cells with regulatory capabilities are moved to the lung microenvironment to participate in the intensified inflammatory response.

## 5. Limitations

The current study had several limitations. It was carried out only in one center on a small population. The reason for this was the inclusion of BALF data in the study protocol and the need to perform VBF in COPD patients for medical reasons and not additionally for the current study. Moreover, we did not perform VBF in the control group for ethical reasons, and, therefore, we could not compare the results in the lung microenvironment to those in the group of COPD patients.

## 6. Conclusions

To summarize, the results of our studies confirm the involvement of NK cells in the inflammatory process in COPD. In COPD, these cells showed a reduced activation state, manifested by a reduced share of functional receptor expression. The decreased expression of activating receptors may be the leading cause of the NK cell population’s dysfunction in COPD. This hypothesis is supported by the observation that increased numbers of immature NK cells were found in both BALF and PB. In BALF, most receptors showed higher expression than NK cell receptors in PB, which may be related to the course of COPD. However, based on immunophenotypic assessment, it cannot be clearly stated whether this is a purely structural or functional change or whether the cells in BALF are activated and ready to take on a cytotoxic function. NK cells in COPD still raise more questions than provide answers in terms of understanding how they regulate their systemic and pulmonary microenvironmental functions.

## Figures and Tables

**Table 1:** Antibodies used in the flow cytometry.

Name	Fluorochrome	Clone	Source
CD45/CD14 (LG-Leucogate)	FITC/PE	2D1/MφP9	BD Biosciences (San Jose, CA, USA)
CD3	PerCP	SK7	BD Biosciences
CD56 (NCAM)	APC	NCAM16.2	BD Biosciences
CD16	PE	D12	BD Biosciences
CD11b (Mac-1)	PE	D12	BD Biosciences
CD27 (TNFRSF7)	FITC	M-T271	BD Biosciences
CD158b (KIR2DL2/DL3)	PE	DX27	Miltenyi Biotec (Bergisch Gladbach, Germany)
CD158i (KIR2DS4)	PE	JJC11.6	Miltenyi Biotec
CD314 (NKG2D)	PE	BAT221	Miltenyi Biotec
CD335 (NKp46)	PE	9E2	Miltenyi Biotec
CD336 (NKp44)	PE	p44-8	BD Biosciences

**Table 2:** Study population characteristics (*n* = 29).

Analyzed Variable	Median	Lower Quartile	Upper Quartile
COPD (*n* = 15)			
Age (years)	67.00	64.00	72.00
BMI (kg/m^2^)	24.00	19.60	31.00
No. of packyears	30.00	20.00	40.00
RV/TLC (%pred)	65.30	61.99	81.14
FEV1 (%pred)	37.00	31.20	47.90
FVC (%pred)	67.00	59.70	75.80
RV (%pred)	172.50	160.00	209.00
DLCO (%pred)	43.00	34.70	67.00
PaO2 (mm Hg)	64.60	61.10	66.70
6MWT (m)	240.00	127.00	390.00
No. of exacerbation/year	2.00	1.00	2.00
Control group (*n* = 14)			
Age (years)	66.00	62.00	70.00
BMI (kg/m^2^)	24.50	23.30	25.70
No. of packyears	25.00	13.00	40.00

Abbreviation: BMI = body mass index, 6MWT = 6 min walking test, DLCO = diffusion capacity of carbon monoxide, FEV1 = forced expiratory volume in one second, FVC = forced vital capacity, PaO2 = arterial oxygen pressure, pred = predicted, RV = residual volume, TLC = total lung capacity.

**Table 3:** The percentage of NK cells among all leukocytes and lymphocytes, and expression markers in PB and BALF in COPD patients group.

	PB		BALF		
	Median	IQR	Median	IQR	*p*-Value
NK %	4.46	4.55	1.46	1.83	0.0234
NK % of lymphocytes	19.90	14.05	33.86	20.6	0.0034
CD27−/CD11b−	65.60	28.30	58.20	54.40	0.8580
CD27+/CD11b−	8.70	7.20	5.50	2.40	0.1523
CD27+/CD11b+	0.90	1.70	11.10	19.80	0.0521
CD27−/CD11b+	16.40	22.30	16.70	28.70	0.5118
CD158b+ %	21.70	21.20	26.00	13.70	0.2104
CD158b+ MFI	1092.00	487.00	850.00	191.00	0.2104
CD158i+ %	0.00	0.40	14.10	10.40	0.0032
CD158i+ MFI	0.00	206.00	863.00	727.00	0.0005
CD314+ %	83.60	17.60	52.50	29.90	0.0019
CD314+ MFI	1168.00	435.00	940.00	281.00	0.5118

Data are presented as median and interquartile range based on Mann–Whitney test. Abbreviation: BALF = Broncho Alveolar Lavage fluid, IQR = interquartile range, MFI = Mean Fluorescence Intensity, NK = natural killer, PB = peripheral blood.

**Table 4:** The percentage of NK cells among all leukocytes and lymphocytes, and expression markers in peripheral blood in COPD and control group.

	COPD		Control Group		
	Median	IQR	Median	IQR	*p*-Value
NK %	4.46	4.55	3.55	1.98	0.555735
NK % of lymphocytes	19.90	14.05	11.19	2.58	0.084723
CD27−/CD11b−	65.60	28.30	20.75	14.00	0.000013
CD27+/CD11b−	8.70	7.20	1.20	1.30	0.000003
CD27+/CD11b+	0.90	1.70	2.85	2.30	0.006833
CD27−/CD11b+	16.40	22.30	76.90	14.40	0.000002
CD158b+ %	21.70	21.20	36.20	23.20	0.002262
CD158b+ MFI	1092.00	487.00	651.50	360.00	0.032770
CD158i+ %	0.00	0.40	5.00	16.50	0.000516
CD158i+ MFI	0.00	206.00	307.00	77.00	0.002676
CD314+ %	83.60	17.60	81.00	10.00	0.651619
CD314+ MFI	1168.0	435.00	807.00	257.00	0.003704

Data are presented as median and interquartile range using Mann–Whitney test. Abbreviation: COPD = Chronic Obstructive Pulmonary Disease, IQR = interquartile range, MFI = Mean Fluorescence Intensity, NK = Natural Killer.

## Data Availability

To get access to the secondary data, please contact the corresponding author.

## References

[1] Agustí A., Melén E., DeMeo D.L., Breyer-Kohansal R., Faner R. (2022). Pathogenesis of Chronic Obstructive Pulmonary Disease: Understanding the Contributions of Gene-Environment Interactions across the Lifespan. Lancet Respir. Med..

[2] Barnes P.J. (2016). Inflammatory Mechanisms in Patients with Chronic Obstructive Pulmonary Disease. J. Allergy Clin. Immunol..

[3] Global Initiative for Chronic Obstructive Lung Disease (GOLD) Global Strategy for the Diagnosis, Management and Prevention of COPD 2015. http://www.goldcopd.org/.

[4] Hogg J.C., Timens W. (2009). The Pathology of Chronic Obstructive Pulmonary Disease. Annu. Rev. Pathol..

[5] (2015). Global, Regional, and National Age-Sex Specific All-Cause and Cause-Specific Mortality for 240 Causes of Death, 1990–2013: A Systematic Analysis for the Global Burden of Disease Study 2013. Lancet.

[6] Hervier B., Russick J., Cremer I., Vieillard V. (2019). NK Cells in the Human Lungs. Front. Immunol..

[7] Cong J., Wei H. (2019). Natural Killer Cells in the Lungs. Front. Immunol..

[8] Marquardt N., Kekäläinen E., Chen P., Kvedaraite E., Wilson J.N., Ivarsson M.A., Mjösberg J., Berglin L., Säfholm J., Manson M.L. (2017). Human Lung Natural Killer Cells Are Predominantly Comprised of Highly Differentiated Hypofunctional CD69−CD56dim Cells. J. Allergy Clin. Immunol..

[9] Kim J.H., Jang Y.J. (2018). Role of Natural Killer Cells in Airway Inflammation. Allergy Asthma Immunol. Res..

[10] Culley F.J. (2009). Natural Killer Cells in Infection and Inflammation of the Lung. Immunology.

[11] Li J., Dong X., Zhao L., Wang X., Wang Y., Yang X., Wang H., Zhao W. (2016). Natural Killer Cells Regulate Th1/Treg and Th17/Treg Balance in Chlamydial Lung Infection. J. Cell Mol. Med..

[12] Hsu A.T., Gottschalk T.A., Tsantikos E., Hibbs M.L. (2021). The Role of Innate Lymphoid Cells in Chronic Respiratory Diseases. Front. Immunol..

[13] Wang J., Urbanowicz R.A., Tighe P.J., Todd I., Corne J.M., Fairclough L.C. (2013). Differential Activation of Killer Cells in the Circulation and the Lung: A Study of Current Smoking Status and Chronic Obstructive Pulmonary Disease (COPD). PLoS ONE.

[14] Global Initiative for Chronic Obstructive Lung Disease (GOLD) (2018). Global Strategy for the Diagnosis, Management, and Prevention of Chronic Obstructive Pulmonary Disease. GOLD. https://goldcopd.org/archived-reports/.

[15] Pinto-Plata V.M., Cote C., Cabral H., Taylor J., Celli B.R. (2004). The 6-Min Walk Distance: Change over Time and Value as a Predictor of Survival in Severe COPD. Eur. Respir. J..

[16] Davis M.D., Walsh B.K., Sittig S.E., Restrepo R.D. (2013). AARC Clinical Practice Guideline: Blood Gas Analysis and Hemoximetry: 2013. Respir. Care.

[17] Du Rand I.A., Blaikley J., Booton R., Chaudhuri N., Gupta V., Khalid S., Mandal S., Martin J., Mills J., Navani N. (2013). British Thoracic Society Guideline for Diagnostic Flexible Bronchoscopy in Adults. Thorax.

[18] Du Rand I.A., Barber P.V., Goldring J., Lewis R.A., Mandal S., Munavvar M., Rintoul R.C., Shah P.L., Singh S., Slade M.G. (2011). British Thoracic Society Guideline for Advanced Diagnostic and Therapeutic Flexible Bronchoscopy in Adults. Thorax.

[19] Miller M.R., Hankinson J., Brusasco V., Burgos F., Casaburi R., Coates A., Crapo R., Enright P., van der Grinten C.P.M., Gustafsson P. (2005). Standardisation of Spirometry. Eur. Respir. J..

[20] Graham B.L., Steenbruggen I., Miller M.R., Barjaktarevic I.Z., Cooper B.G., Hall G.L., Hallstrand T.S., Kaminsky D.A., McCarthy K., McCormack M.C. (2019). Standardization of Spirometry 2019 Update. An Official American Thoracic Society and European Respiratory Society Technical Statement. Am. J. Respir. Crit. Care Med..

[21] Klech H. (1989). Technical Recomendations and Guidelines for Bronchoalveolar Lavage (BAL). Report of the European Society of Pneumonology Task Group on BAL. Eur. Respir. J..

[22] Meyer K.C., Raghu G., Baughman R.P., Brown K.K., Costabel U., Du Bois R.M., Drent M., Haslam P.L., Kim D.S., Nagai S. (2012). An Official American Thoracic Society Clinical Practice Guideline: The Clinical Utility of Bronchoalveolar Lavage Cellular Analysis in Interstitial Lung Disease. Am. J. Respir. Crit. Care Med..

[23] Baughman R.P. (2007). Technical Aspects of Bronchoalveolar Lavage: Recommendations for a Standard Procedure. Semin. Respir. Crit. Care Med..

[24] Vivier E., Tomasello E., Baratin M., Walzer T., Ugolini S. (2008). Functions of Natural Killer Cells. Nat. Immunol..

[25] Bu T., Wang L.F., Yin Y.Q. (2020). How Do Innate Immune Cells Contribute to Airway Remodeling in Copd Progression?. Int. J. COPD.

[26] Eriksson Ström J., Pourazar J., Linder R., Blomberg A., Lindberg A., Bucht A., Behndig A.F. (2018). Cytotoxic Lymphocytes in COPD Airways: Increased NK Cells Associated with Disease, INKT and NKT-like Cells with Current Smoking. Respir. Res..

[27] Urbanowicz R.A., Lamb J.R., Todd I., Corne J.M., Fairclough L.C. (2010). Enhanced Effector Function of Cytotoxic Cells in the Induced Sputum of COPD Patients. Respir. Res..

[28] Tang Y., Li X., Wang M., Zou Q., Zhao S., Sun B., Xu L., Jiang Y. (2013). Increased Numbers of NK Cells, NKT-Like Cells, and NK Inhibitory Receptors in Peripheral Blood of Patients with Chronic Obstructive Pulmonary Disease. Clin. Dev. Immunol..

[29] Andersson A., Malmhäll C., Houltz B., Tengvall S., Sjöstrand M., Qvarfordt I., Lindén A., Bossios A. (2016). Interleukin-16-Producing NK Cells and T-Cells in the Blood of Tobacco Smokers with and without COPD. Int. J. COPD.

[30] Theresine M., Patil N.D., Zimmer J. (2020). Airway Natural Killer Cells and Bacteria in Health and Disease. Front. Immunol..

[31] Qiu F., Liang C.L., Liu H., Zeng Y.Q., Hou S., Huang S., Lai X., Dai Z. (2017). Impacts of cigarette smoking on immune responsiveness: Up and down or upside down?. Oncotarget..

[32] Pezzuto A., Ricci A., D’Ascanio M., Moretta A., Tonini G., Calabrò N., Minoia V., Pacini A., De Paolis G., Chichi E. (2023). Short-Term Benefits of Smoking Cessation Improve Respiratory Function and Metabolism in Smokers. Int. J. Chron. Obstruct. Pulmon. Dis..

[33] Ioka A., Nakamura M., Shirokawa N., Kinoshita T., Masui S., Imai K., Nakachi K., Oshima A. (2001). Natural killer activity and its changes among participants in a smoking cessation intervention program--a prospective pilot study of 6 months’ duration. J. Epidemiol..

[34] Motz G.T., Eppert B.L., Wortham B.W., Amos-Kroohs R.M., Flury J.L., Wesselkamper S.C., Borchers M.T. (2010). Chronic cigarette smoke exposure primes NK cell activation in a mouse model of chronic obstructive pulmonary disease. J. Immunol..

[35] Chen Y.-C., Lin M.-C., Lee C.-H., Liu S.-F., Wang C.-C., Fang W.-F., Chao T.-Y., Wu C.-C., Wei Y.-F., Chang H.-C. (2018). Defective Formyl Peptide Receptor 2/3 and Annexin A1 Expressions Associated with M2a Polarization of Blood Immune Cells in Patients with Chronic Obstructive Pulmonary Disease. J. Transl. Med..

[36] Urbanowicz R.A., Lamb J.R., Todd I., Corne J.M., Fairclough L.C. (2009). Altered Effector Function of Peripheral Cytotoxic Cells in COPD. Respir. Res..

[37] Hodge G., Mukaro V., Holmes M., Reynolds P.N., Hodge S. (2013). Enhanced Cytotoxic Function of Natural Killer and Natural Killer T-like Cells Associated with Decreased CD94 (Kp43) in the Chronic Obstructive Pulmonary Disease Airway. Respirology.

[38] Cooper M.A., Fehniger T.A., Turner S.C., Chen K.S., Ghaheri B.A., Ghayur T., Carson W.E., Caligiuri M.A. (2001). Human Natural Killer Cells: A Unique Innate Immunoregulatory Role for the CD56 Bright Subset. Blood..

[39] Fauriat C., Long E.O., Ljunggren H.-G., Bryceson Y.T. (2010). Regulation of Human NK-Cell Cytokine and Chemokine Production by Target Cell Recognition. Blood.

[40] Silva A., Andrews D.M., Brooks A.G., Smyth M.J., Hayakawa Y. (2008). Application of CD27 as a Marker for Distinguishing Human NK Cell Subsets. Int. Immunol..

[41] Fu B., Wang F., Sun R., Ling B., Tian Z., Wei H. (2011). CD11b and CD27 Reflect Distinct Population and Functional Specialization in Human Natural Killer Cells. Immunology..

[42] Freeman C.M., Stolberg V.R., Crudgington S., Martinez F.J., Han M.-K., Chensue S.W., Arenberg D.A., Meldrum C.A., McCloskey L., Curtis J.L. (2014). Human CD56+ Cytotoxic Lung Lymphocytes Kill Autologous Lung Cells in Chronic Obstructive Pulmonary Disease. PLoS ONE.

[43] Finch D.K., Stolberg V.R., Ferguson J., Alikaj H., Kady M.R., Richmond B.W., Polosukhin V.V., Blackwell T.S., McCloskey L., Curtis J.L. (2018). Lung Dendritic Cells Drive Natural Killer Cytotoxicity in Chronic Obstructive Pulmonary Disease via IL-15Ra. Am. J. Respir. Crit. Care Med..

[44] Caligiuri M.A. (2008). Human Natural Killer Cells. Blood.

[45] Mistry A.R., O’Callaghan C.A. (2007). Regulation of Ligands for the Activating Receptor NKG2D. Immunology.

[46] Lee J., Taneja V., Vassallo R. (2012). Cigarette Smoking and Inflammation: Cellular and Molecular Mechanisms. J. Dent. Res..

[47] Elisia I., Lam V., Cho B., Hay M., Li M.Y., Yeung M., Bu L., Jia W., Norton N., Lam S. (2020). The Effect of Smoking on Chronic Inflammation, Immune Function and Blood Cell Composition. Sci. Rep..

[48] Rascle P., Woolley G., Jost S., Manickam C., Reeves R.K. (2023). NK Cell Education: Physiological and Pathological Influences. Front. Immunol..

[49] Dębska-Zielkowska J., Moszkowska G., Zieliński M., Zielińska H., Dukat-Mazurek A., Trzonkowski P., Stefańska K. (2021). Kir Receptors as Key Regulators of Nk Cells Activity in Health and Disease. Cells.

[50] Qi C., Liu Q. (2023). Natural Killer Cells in Aging and Age-Related Diseases. Neurobiol. Dis..

[51] Shehata H.M., Hoebe K., Chougnet C.A. (2015). The Aged Nonhematopoietic Environment Impairs Natural Killer Cell Maturation and Function. Aging Cell..

[52] Chiu B.C., Martin B.E., Stolberg V.R., Chensue S.W. (2013). The Host Environment Is Responsible for Aging-Related Functional NK Cell Deficiency. J. Immunol..

[53] Kaczmarek M., Wasicka K., Tin-Tsen Chou J., Popowicz P., Rzetelska Z., Łagiedo-Żelazowska M., Piwowarczyk K., Leszczyńska M. (2020). NK Cells in Patients with Chronic Rhinosinusitis Show Decreased Maturity and Limited Expression of Functional Receptors. Immunobiology.

[54] Tang J., Ramis-Cabrer D., Curull V., Wang X., Qin L., Mateu-Jiménez M., Duran X., Pijuan L., Rodríguez-Fuster A., Espases R.A. (2020). Immune Cell Subtypes and Cytokines in Lung Tumor Microenvironment: Influence of COPD. Cancers.

[55] Rao Y., Le Y., Xiong J., Pei Y., Sun Y. (2021). NK Cells in the Pathogenesis of Chronic Obstructive Pulmonary Disease. Front. Immunol..

[56] Pascual-Guardia S., Ataya M., Ramírez-Martínez I., Yélamos J., Chalela R., Bellido S., López-Botet M., Gea J. (2020). Adaptive NKG2C+ Natural Killer Cells Are Related to Exacerbations and Nutritional Abnormalities in COPD Patients. Respir. Res..

[57] Global Initiative for Chronic Obstructive Lung Disease (GOLD) (2023). Global Strategy for the Diagnosis, Management, and Prevention of Chronic Obstructive Pulmonary Disease. GOLD. https://goldcopd.org/archived-reports/.

[58] Hong X., Xiao Z. (2023). Changes in Peripheral Blood TBNK Lymphocyte Subsets and Their Association with Acute Exacerbation of Chronic Obstructive Pulmonary Disease. J. Int. Med. Res..

[59] Ryu M.H., Yun J.H., Morrow J.D., Saferali A., Castaldi P., Chase R., Stav M., Xu Z., Barjaktarevic I., Han M.L. (2023). Blood Gene Expression and Immune Cell Subtypes Associated with Chronic Obstructive Pulmonary Disease Exacerbations. Am. J. Respir. Crit. Care Med..

[60] Pichavant M., Sharan R., Le Rouzic O., Olivier C., Hennegrave F., Rémy G., Pérez-Cruz M., Koné B., Gosset P., Just N. (2015). IL-22 Defect During Streptococcus Pneumoniae Infection Triggers Exacerbation of Chronic Obstructive Pulmonary Disease. EBioMedicine.

